# Overload of Glucose Metabolism as Initiating Factor in Diabetic Embryopathy and Prevention by Glyoxalase 1 Inducer Dietary Supplement

**DOI:** 10.3390/antiox14081022

**Published:** 2025-08-21

**Authors:** Parri Wentzel, Mingzhan Xue, Naila Rabbani, Ulf J. Eriksson, Paul J. Thornalley

**Affiliations:** 1 Department of Medical Cell Biology, Biomedical Center, Uppsala University, 75123 Uppsala, Sweden; parri.wentzel@mcb.uu.se; 2 Diabetes Research Center, Qatar Biomedical Research Institute, Hamad Bin Khalifa University, Qatar Foundation, Doha P.O. Box 34110, Qatar; mxue@hbku.edu.qa; 3 QU (Qatar University) Health, Qatar University, University Street, Doha P.O. Box 2713, Qatar; nailarabbani65@gmail.com; 4 College of Health and Life Sciences, Hamad Bin Khalifa University, Qatar Foundation, Doha P.O. Box 34110, Qatar

**Keywords:** hyperglycemia, metabolic dysfunction, glycation, diabetic embryopathy, teratogenesis

## Abstract

Hyperglycemia in early-stage embryogenesis is linked to diabetic embryopathy. High-glucose-concentration-induced accumulation of hexokinase-2 (HK2) may initiate metabolic dysfunction that contributes to diabetic embryopathy, including increased formation of methylglyoxal (MG). In this study, we evaluated changes in HK2 protein levels and embryo dysmorphogenesis in an experimental model of diabetic embryopathy. Rat embryos were cultured with high glucose concentrations, and the effects of glyoxalase 1 (Glo1) inducer, *trans*-resveratrol and hesperetin (tRES + HESP) were evaluated. Rat embryos, on gestational day 9, were cultured for 48 h in low and high glucose concentrations with or without tRES + HESP. Embryo crown–rump length, somite number, malformation score, concentrations of HK2 and Glo1 protein, rates of glucose consumption, and MG formation were assessed. Under low-glucose conditions, embryos exhibited normal morphogenesis. In contrast, high-glucose conditions led to reduced crown–rump length and somite number, and an increased malformation score. The addition of 10 μM tRES + HESP reversed these high glucose-induced changes by 60%, 49%, and 47%, respectively. Embryos cultured in high glucose showed increases in HK2 concentration (42%), glucose consumption (75%), and MG formation (27%), normalized to embryo volume. These elevated HK2 levels were normalized by treatment with 10 μM tRES + HESP. Thus, high-glucose-induced metabolic dysfunction and embryopathy may both be initiated by HK2 accumulation and may be preventable with tRES + HESP treatment.

## 1. Introduction

Diabetic embryopathy is defined as the development of congenital malformations in the offspring of mothers with diabetes [1,2,3,4,5]. Offspring of women with type 1 or type 2 diabetes mellitus, or those with obesity, are at an increased risk of developing congenital malformations [6]. These malformations arise before the seventh week of gestation [7]. The prevalence of congenital malformations in pregnancies of women with diabetes is approximately 5–6% [8,9]. Embryo malformations occur in the earliest stages of organogenesis and may affect the brain and spinal cord, heart, kidneys, gut, and skeletal muscles, leading to pre- or postnatal mortality or disability [7,10]. The risk of malformations is linked to glycated hemoglobin A1C, suggesting that hyperglycemia and the quality of glycemic control are involved in the pathogenesis [11,12,13].

Diabetic embryopathy may be modeled using in vivo experimental diabetes or by in vitro culture of rat embryos in high glucose concentrations [14,15,16]. In laboratory rodents, experimental diabetic embryopathy is characterized by reduced crown–rump length, decreased somite number, open neural tubes, rotational defects, and/or cardiac enlargement [15,17]. Increased glucose metabolism is required for the development of experimental diabetic embryopathy [18,19,20,21]. The metabolic dysfunction contributing to the pathogenesis of high-glucose-induced embryopathy is characterized by increased formation of reactive oxygen species (ROS) [22,23], elevated hexosamine pathway activity [24,25], altered protein kinase C (PKC) activity [26,27], increased production of reactive dicarbonyl glycating intermediates such as methylglyoxal (MG) [28], and endoplasmic reticulum (ER) stress [29,30,31]. A hypothesis suggests that hexokinase-2 (HK2)-linked unscheduled glycolysis and glycolytic overload may offer new insights into the metabolic dysfunction in diabetic embryopathy. According to the hypothesis, the abnormally high flux of glucose metabolism seen in hyperglycemia results from the stabilization of HK2 against proteolysis by high cellular glucose concentrations [32].

In early-stage rat embryogenesis, glucose enters the embryo by GLUT1 and GLUT3 glucose transporters. GLUT1 was found mainly in neuroepithelial cells and microvessels surrounding the neural tube, with expression maintained during incubation of conceptuses in high glucose concentrations [33]. Cellular glucose concentration was increased in the embryo neuroectoderm of streptozotocin (STZ)-induced diabetic rats at days 11–12 of pregnancy; the respective values were 27–37 mM in embryos of STZ diabetic rats, compared to 7–8 mM in embryos of healthy controls [34]. Hexokinases (HKs) are expressed throughout embryo development. HK1 and HK2 were found from early-stage rat embryogenesis, with HK3 and HK4 or glucokinase expression occurring later, from days 15 to 18 of gestation [35]. HK1 and HK2 have a high affinity for glucose substrate with enzyme kinetics saturated by 99% and 96%, respectively, in normoglycemia (5 mM glucose) [32,36]. Given this high rate of enzyme saturation, it was surprising that the concentrations of early-stage glycolytic intermediates, glucose-6-phosphate (G6P) and fructose-6-phosphate (F6P), were increased two-fold in rat embryos incubated in high glucose concentration, normally indicative of increased flux of glucose metabolism through early-stage glycolysis [37]. From our recent research findings [32,38], this conundrum may be explained by an increase in in situ activity of hexokinase produced by stabilization of HK2 to proteolysis in a high cellular glucose concentration. This provides for an increased flux of glucose metabolism through glycolysis in the embryo, sustaining abnormally high concentrations of early-stage glycolytic intermediates.

Herein, we employed an experimental model of diabetic embryopathy: we observed rat embryos cultured in high glucose concentration, assessing the effects on embryo dysmorphogenesis. For the first time, we studied the possible link of embryo dysmorphogenesis to increased embryonic concentration of HK2 protein and metabolic dysfunction, as indicated by increased formation of MG. To further explore a possible functional link, we assessed the effect of treatment with a glyoxalase 1 (Glo1) inducer, equimolar *trans*-resveratrol, and hesperetin combination (tRES + HESP). tRES + HESP is an optimized inducer of the expression of Glo1, the major enzyme catalyzing the metabolism of MG, increasing Glo1 expression through the activation of transcription factor nuclear factor erythroid 2-related factor 2 (Nrf2) [39]. It was also found to prevent HK2-linked glycolytic overload in primary cultures of endothelial cells and fibroblasts by induction of expression of glucose-6-phosphate dehydrogenase (G6PD), decreasing G6P/Mondo A/Mlx-dependent transcription of HK2, thereby decreasing protein and activity of HK2 to levels found in normoglycemia [38]. Our present work is a proof-of-concept study where initial findings suggest that treatment with tRES + HESP produces therapeutic benefit in the experimental model at concentrations that are likely clinical translatable.

## 2. Methods

### 2.1. Rat Whole-Embryo Culture

Female and male outbred Sprague–Dawley rats were housed and maintained at the Biomedical Center, Uppsala University, Uppsala, Sweden. They had free access to a commercial pelleted diet (R 36; Analycen, Lidköping, Sweden) and tap water. For rat housing, the ambient temperature was 22 °C and a 12 h light/dark cycle was maintained. Female and male rats were caged together during the night for conception. We verified conception by the presence of sperms in a vaginal smear. The morning of the day of conception was designated gestational day 0. Pregnant rats were killed by cervical dislocation between 11 am and 1 pm on gestational day 9 and the embryos were recovered. The number of embryos per pregnant rat was about 10–12 in this study. The conceptuses were explanted and whole embryos were cultured by methods we described previously [17,40]. The somite number of the day 9 embryos were counted and embryos with 3–4 somites were used to start the cultures. Embryos were maintained in Falcon 2070 polypropylene tubes in a roller incubator at 38 °C and 60 rpm. They were within their intact yolk sacs at this stage. There were four conceptuses per tube cultured in a 4 mL medium (80% *v/v* rat serum and 20% *v/v* isotonic saline). We collected arterial blood from rats. After immediate centrifugation, serum was prepared and supplemented with sodium benzylpenicillinate (60 µg/mL) and streptomycin (100 µg/mL). Serum was stored frozen and was heat-inactivated at 56 °C for 1 h immediately before use. The final glucose concentrations were 10 and 30 mM (standard euglycemic and hyperglycemic conditions for this experimental model [28,41,42,43]) with and without 5, 10 and 20 µM tRES + HESP. Tubes were filled with an atmosphere of 5% O_2_, 5% CO_2_, and 90% N_2_ (*v/v/v*), capped tightly, and incubated at 37 °C for 24 h. Conceptuses were then transferred to new culture tubes with a new medium and filled with an atmosphere of 20% O_2_, 5% CO_2_, and 75% N_2_ (*v/v/v*) and cultured for 20 h. The tubes were then placed under an atmosphere of 40% O_2_, 5% CO_2_, and 55% N_2_ (*v/v/v*) for 10 min and the embryos were collected after a further 4—6 h. The total period of culture was 48 h. At the time of collection, the gestational day was 11.7. For experimental examination, conceptuses were placed in Petri dishes containing isotonic saline. Viewing the conceptuses under a stereo microscope (magnification 10–20), we used gentle dissection to separate the embryo from the corresponding yolk sac and amniotic membranes. The embryos were then immediately snap-frozen in separate tubes and stored at −80 °C until analysis.

**Figure 1:**
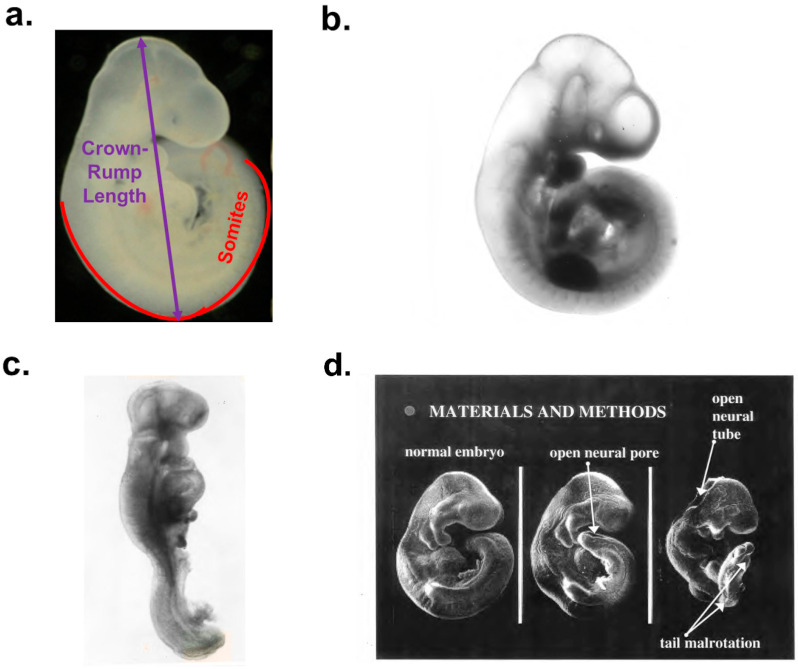
Assessment of development in rat embryos after exposure to 10 or 30 mM glucose in whole-embryo culture. (**a**) Normal embryo, fully rotated, with crown–rump length 3.6 mm, 28 somites and malformation score 0 after 48 h culture in 10 mM glucose. Note assessment of crown–rump length and somites. Rat embryo morphology viewed in stereo microscope at magnification of 10–20×. (**b**) Normal embryo, fully rotated with crown–rump length 3.5 mm, 29 somites and malformation score 0 after culture in 10 mM glucose. Rat embryo morphology viewed in stereo microscope at magnification of 10–20×. (**c**). Malformed embryos, malrotated with open neural tube and maldeveloped heart after 48 h culture in 30 mM glucose. Estimated crown–rump length 1.9 mm, 20 somites and malformation score 10. Rat embryo morphology viewed in stereo microscope at magnification of 10–20×. (**d**) Electron micrographs of 3 embryos after culture in 30 mM glucose. Left: normal embryo (crown–rump length 3.5 mm, 29 somites, malformation score 0. Center: embryo with one malformation, an open neural pore, crown–rump length of 3.4 mm, 29 somites, and malformation score of 1. Right: embryo with multiple malformations, open neural tube and tail malrotation, crown–rump length of 1.6 mm, 13 somites, and malformation score of 10.

Embryo dysmorphogenesis was quantified by measuring the crown–rump length and counting the number of somites. We classified embryos as morphologically normal or with malformations of differing severity. The morphological score was deduced. This was the average value for each experimental condition. The morphological scores allocated were as follows: 0, embryos with normal morphology—fully rotated with a closed neural tube; 1, embryos with minor malformation—one minor difference from normal morphology, often containing the neural tube with an open posterior end; 5, embryos with moderate malformation—one major malformation, which was often a slight tail twist or open neural tube in the rhombencephalon area; and 10, embryos with severe malformations—an embryo with multiple major malformations (open neural tube, rotational defects, and/or heart enlargement). These are illustrated in (Figure 1a–d).

### 2.2. Western Blotting

For Western Blotting, embryo protein extracts were prepared with a radio-immunoprecipitation assay (RIPA) buffer with a protease and phosphatase inhibitor cocktail (cat no 9806; Cell Signaling Technology, Leiden, The Netherlands). A detergent-compatible protein assay kit was used to determine protein concentration (DC protein assay kit II, cat no 5000112, Bio-Rad, Watford, UK). Embryo protein extracts (20 µg) were loaded to SDS/PAGE 10% polyacrylamide gels. After electrophoresis, the proteins were transferred to a PVDF membrane and the membrane was blocked with 5% (*w/v*) non-fat dried skimmed milk powder in Tris-buffered saline (TBST; 10 mM Tris/HCl, pH 7.5, 150 mM NaCl and 0.05% Tween 20). The membrane was incubated with primary antibodies at 4 °C overnight. The anti-HK2 antibody was a polyclonal rabbit IgG (1:5000 dilution; cat no PA5-29326; Invitrogen, Carlsbad, CA, USA) and the anti-Glo1 antibody was a polyclonal rabbit IgG (1:4000 dilution; prepared and purified in-house) [38]. The reference protein β-actin was probed with an anti-β-actin antibody (polyclonal rabbit IgG, cat no 4967, 1:1000 dilution, Cell Signalling Biotechnology, Danvers, MA, USA). After washing with TBST, the membrane was incubated with the appropriate secondary antibody–horseradish peroxidase conjugate for 1 h at room temperature (goat anti-rabbit IgG–horseradish peroxidase conjugate for HK2, GLO1, and ACTB primary antibodies, 1:8000 dilution, cat no A9169, Merck, Poole, Dorset, UK). Immunoreactivity was detected using enhanced chemiluminescence (ECL) and visualized with the ChemiDoc^TM^MP Imaging System (Bio-Rad Laboratories, Hercules, CA, USA). The intensities of protein bands were quantified by the software ImageQuant TL 12.5 (GE Healthcare, Amersham, Buckinghamshire, UK). Estimates of embryonic concentrations of HK2 and Glo1 per unit embryo volume were required. A correction factor for decreased embryo volume in 30 mM glucose was applied to HK2/β-actin and Glo1/β-actin band intensity ratios, proportional to the amounts of embryonic HK2 and Glo1 protein. A surrogate measure of embryo volume is crown–rump length, which correlates strongly with rat embryo weight (and embryo volume) over a similar gestational period, as studied herein (gestational days 10–12, r = 0.98 [44]). The correction factor applied was the crown–rump length ratio in low/high-glucose-concentration cultures.

### 2.3. Metabolic Flux Measurements

Flux of glucose consumption and formation of MG during the 2-day culture was determined. Flux of MG formation was conveniently quantified as increased concentration of the terminal glyoxalase pathway metabolite, D-lactate [38]. Measurements were made of the culture medium at the start and end of day 1 and day 2. Fluxes of glucose consumption and D-lactate formation were deduced by addition of estimates of the amount of glucose consumed or D-lactate formed on day 1 and day 2. 

Glucose concentration was assayed using the HK method using a commercial kit (cat no GAHK20, Merck). Glucose was converted to G6P by HK in the presence of non-limiting ATP, and the G6P formed was converted to 6-phospho-D-glucono-1,5-lactone by bacterial G6PD in the presence of non-limiting NAD^+^, with the amount of glucose present determined from equimolar amount of NADH, and quantified by microplate absorbance spectrophotometry at 340 nm. Assay mixtures contained the following: 1.5 mM NAD^+^, 1 mM ATP, 1 unit/mL HK, and 1 unit/mL G6PD. A standard curve was constructed in the range of 0.25–1.50 mM glucose. An aliquot of standard or diluted sample (25 µL) was added to each well of a microplate, and then the assay reagent (225 µL) was added. The plate was incubated at room temperature for 15 min before the absorbance at 340 nm was read and the concentration of glucose was deduced via interpolation on the standard curve.

The concentrations of D-lactate in culture media were assayed by an end-point enzymatic fluorometric assay with D-lactic dehydrogenase from *Staphylococcus epidermidis* (cat no L9636, Merck). D-Lactate was converted to pyruvate in the presence of D-lactic dehydrogenase and non-limiting NAD^+^, forming NADH, with excess hydrazine hydrate added to drive the forward reaction to completion. The amount of D-lactate present was deduced from the equimolar NADH formed, quantified by microplate fluorimetry, with an excitation wavelength of 340 nm and an emission wavelength of 460 nm. Culture media samples were deproteinized beforehand with perchloric acid (PCA). Briefly, an aliquot of ice-cold PCA (1 mL, 0.6 M) was added to the media (500 μL) to deproteinize samples. The mixture was vortexed and left on ice for 10 min as the protein precipitation concluded. The samples were centrifuged (7000× *g*, 4 °C, 5 min) to sediment the precipitate. An aliquot of the supernatant (700 µL) was neutralized to pH 7 through the addition of potassium bicarbonate (200 µL, 2 M). Samples were vortexed and centrifuged again to sediment the potassium perchlorate precipitate. An aliquot of supernatant or D-lactate standard solution (100 µL) was added in duplicate to the 96-well black microplate containing glycine hydrazine buffer (100 µL; 1.2 M glycine, 0.5 M hydrazine hydrate, 2.5 mM diethylenetriaminepentaacetic acid, pH 9.2) and NAD^+^ (4 mM, 25 µL). The reaction was started through the addition of D-lactic dehydrogenase enzyme (25 µL, 250 U/mL). The microplate was protected from light by being wrapped in aluminum foil and incubated at 37 °C in the dark for 2 h. Each sample had its own blank: incubation mixture without the addition of an enzyme. A standard curve was prepared by assaying of the D-lactate standard (0.5–3 nmol D-lactate, sodium salt; cat no 71716, Merck) and the amount of D-lactate present in the samples was deduced via interpolation on the calibration curve.

Estimates of metabolic fluxes per unit embryo volume were required. A correction factor for decreased embryo volume was applied using the crown–rump length ratio in low/high-glucose-concentration cultures, as for the Western Blotting method described above.

### 2.4. Statistical Analyses

Data are mean ± SEM of a minimum of 3 independent biological replicates. Significance differences were assessed by Student’s *t*-test (2 groups) and one-way analysis of variance (ANOVA; 7 groups).

## 3. Results

### 3.1. Dysmorphogenesis of Rat Embryo in Culture Induced by High Glucose Concentration: Effect of Glyoxalase 1 Inducer

**Figure 2:**
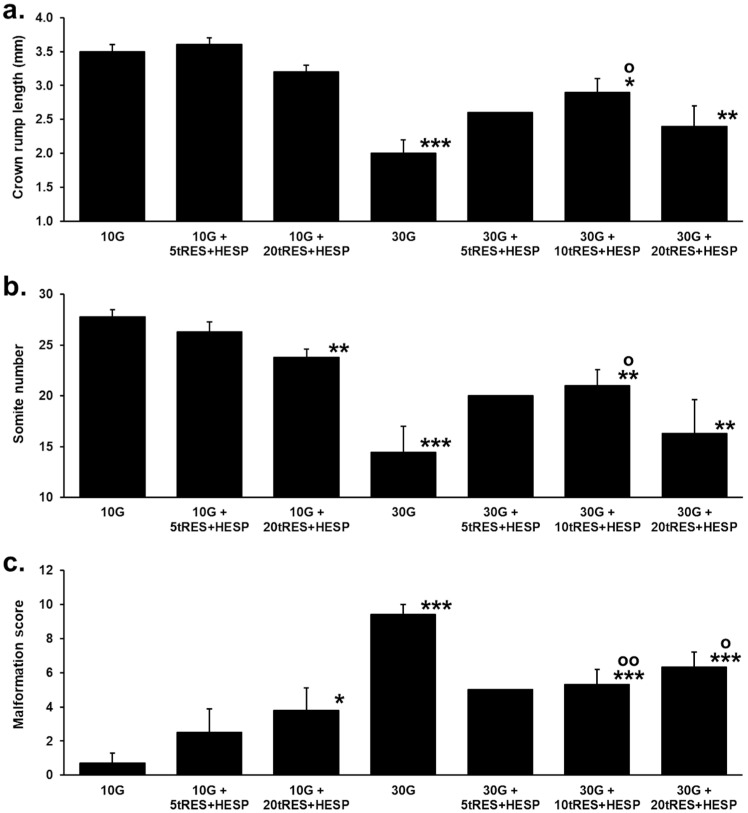
Effect of glucose and Glyoxalase 1 inducer on embryogenesis in vitro. (**a**) Crown–rump length. (**b**) Somite number. (**c**) Malformation score. Key: 10 G, +10 mM glucose (*n* = 9); 10 G + 5tRES + HESP, +10 mM glucose and 5 µM tRES + HESP (*n* = 4); 10 G + 20tRES + HESP, +10 mM glucose and 20 µM tRES + HESP (*n* = 4); 30 G, +30 mM glucose (*n* = 9); 30 G + 5tRES + HESP, +30 mM glucose and 5 µM tRES + HESP (*n* = 2); 30 G + 10tRES + HESP, +30 mM glucose and 10 µM tRES + HESP (*n* = 16); and 30 G + 20tRES + HESP, +30 mM glucose and 20 µM tRES + HESP (*n* = 8). Data are mean ± SEM except mean only where *n* = 2. Significance: crown–rump length, *p* < 0.001, somite number, *p* < 0.01 and malformation score, *p* < 0.001 (*one-way ANOVA for all 7 groups*); *, **, and ***, *p* < 0.05, *p* < 0.01, and *p* < 0.001 with respect to low-glucose-concentration (10 mM) control; and o and oo, *p* < 0.05 and *p* < 0.01 with respect to high-glucose-concentration (30 mM) control (*Student’s t*-test).

Cultures of rat embryos in 10 mM glucose produced normal embryos with a crown–rump length of 3.5 mm, with 28 somites, and a malformation score of 0.7. There were no significant changes in embryo malformation scores with the addition of 5 µM tRES + HESP to low-glucose-concentration cultures. There was a modest decrease in the somite number (−14%) and five-fold increase in the malformation score with the addition of 20 µM tRES + HESP to low-glucose-concentration cultures, suggesting this high concentration of tRES + HESP may induce embryo malformation. Cultures of rat embryos in the 30 mM glucose concentration mimicking hyperglycemia found in diabetes decreased crown–rump length to 2.0 mm (−41%) and somite number to 14 (−48%), and increased the malformation score to 9.4 (14-fold). There was an apparent increased crown–rump length and somite number and decreased malformation score with the addition of 5 µM tRES + HESP to high-glucose-concentration cultures, compared with low-glucose cultures. Only two replicates were successfully completed for this treatment; one sample was lost during sample collection, so we were unable to assess the statistical significance of the apparent changes. An addition of 10 µM tRES + HESP to high-glucose-concentration cultures increased crown–rump length to 2.9 mm, increased somite number to 21, and decreased the malformation score to 5.3, reversing these high-glucose-concentration-induced changes by 60%, 49%, and 47%, respectively. Surprisingly, the addition of 20 µM tRES + HESP to high-glucose-concentration cultures did not increase crown–rump length or somite number compared to high-glucose-concentration control, whereas there was a minor decrease in malformation score (−38%) (Figure 2a–c).

### 3.2. Embryonic Hexokinase-2 and Glyoxalase 1 Concentration in Model Hyperglycemia

**Figure 3:**
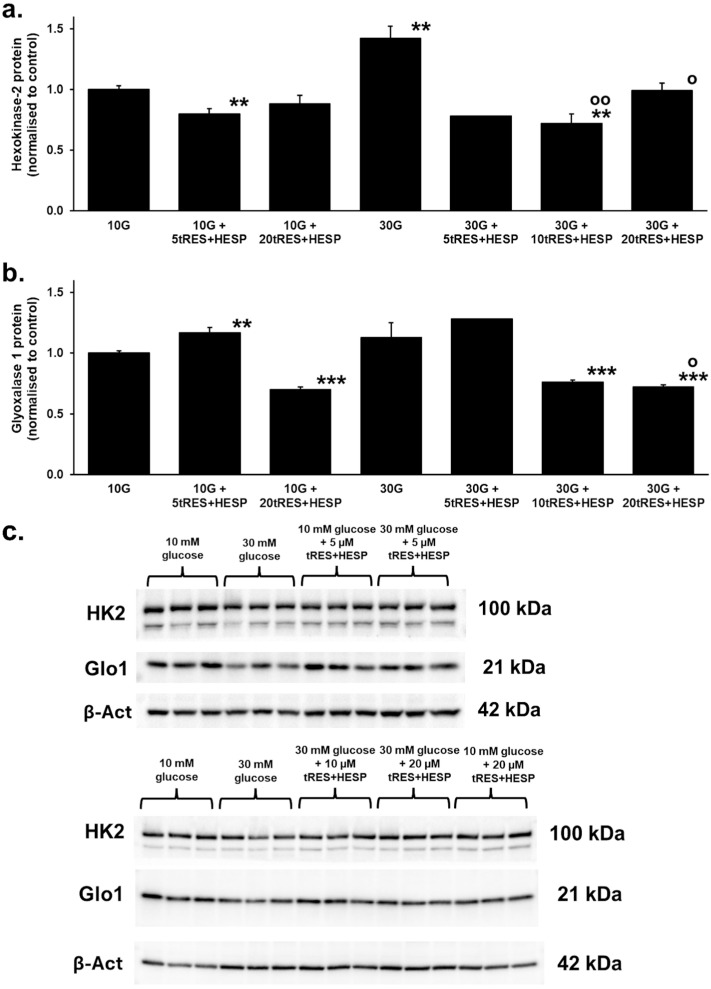
Effect of glucose and Glyoxalase 1 inducer on hexokinase-2 and glyoxalase 1 concentration in embryogenesis in vitro. (**a**) Hexokinase 1 protein. (**b**) Glyoxalase 1 protein. The sample key is the same as given for Figure 2: Replicates: for HK2 protein and Glo1 protein measurements, 10 G (*n* = 6), 10 G + 5tRES + HESP and 10 G + 20tRES + HESP (*n* = 3), 30 G (*n* = 6), 30 G + 5tRES + HESP (*n* = 2), and 30 G + 10tRES + HESP and 30 G + 10tRES + HESP (*n* = 3). Data are mean ± SEM (*n*) except mean only where *n* = 2 and corrected for the decrease in embryo volume, assuming the change in embryo volume is proportional to the change in crown–rump length (Figure 1). Significance: HK2 and Glo1, *p* < 0.001 (*one-way ANOVA for all 7 groups*); ** and ***, *p* < 0.01, and *p* < 0.001 with respect to low-glucose-concentration (10 mM) control; and o and oo, *p* < 0.05 and *p* < 0.01 with respect to high-glucose-concentration (30 mM) control (*Student’s t*-test). (**c**) Western Blotting images used for densitometry showing immunoblotting bands for hexokinase 2 (HK2), glyoxalase 1 (Glo1), and β-actin housekeeping protein (β-Act).

Previous studies of human endothelial cells and fibroblasts in vitro found increased cellular concentrations of the HK2 protein in high glucose concentrations which were corrected by treatment with tRES + HESP [38,45]. We quantified the embryo concentrations of HK2 and Glo1 protein by Western Blotting on day two of culture. For HK2, we found that HK2 protein concentration in 10 mM glucose cultures decreased by 20% through the addition of 5 µM tRES + HESP and did not change significantly through the addition of 20 µM tRES + HESP. In 30 mM glucose cultures, however, HK2 protein concentrations increased by 42%. Interestingly, treatment with 5 µM, 10 µM, and 20 µM tRES + HESP reversed the increase in HK2 in high-glucose-concentration cultures, although with 5 µM tRES + HESP, data were available from only two replicates (Figure 3a).

Previous studies of human endothelial cells and fibroblasts in vitro found decreased cellular concentrations of Glo1 in high glucose concentrations which were corrected by treatment with 10 µM tRES + HESP [38,45]. In rat embryo cultures in 10 mM glucose, the addition of 5 µM tRES + HESP increased Glo1 protein concentration by 17%, whereas the addition of 20 µM tRES + HESP decreased Glo1 protein concentration by 30%. In embryo cultures with 30 mM glucose, the concentration of Glo1 protein was not changed significantly. Interestingly, for treatment of high glucose concentration cultures with 5 µM tRES + HESP, there was a suggestion of increased Glo1 protein concentration by 28%, although data were available from only two replicates. For treatment of high-glucose-concentration cultures with 10 µM and 20 µM tRES + HESP, the Glo1 protein concentration was decreased by 24% and 28%, respectively, compared to 10 mM glucose cultures (Figure 3b).

### 3.3. Embryonic Consumption of Glucose and Formation of Methylglyoxal in Model Hyperglycemia

Given the increase in concentration of the HK2 protein in embryos in a high glucose concentration, we measured the glucose consumption by embryos in low- and high-glucose-concentration cultures in the 2-day culture. The flux of glucose consumption in high-glucose-concentration cultures increased by 75% compared to low-glucose-concentration cultures per unit embryo volume. Glucose consumption values are as follows (µmol/embryo/day): 10 mM glucose, 5.12 ± 0.04; and 30 mM glucose, 8.93 ± 0.25, *p* < 0.001 (*n* = 3).

We also explored if the formation of D-lactate, a characteristic of glycolytic overload [38], was increased. The flux of formation of D-lactate in high-glucose-concentration cultures was increased by 27% per unit embryo volume compared to low-glucose-concentration cultures. D-Lactate formation values are as follows (nmol/embryo/day): 10 mM glucose, 25.6 ± 1.2; and 30 mM glucose, 32.4 ± 2.1, *p* < 0.05 (*n* = 3).

## 4. Discussion

Herein we provide evidence from the study of rat embryos in high-glucose-concentration cultures as a model of diabetic embryopathy that metabolic dysfunction-driving embryo dysmorphogenesis therein is initiated by HK2-linked unscheduled glycolysis and glycolytic overload [32]. Cultures of rat embryos in 30 mM glucose from approximately gestational days 9 to 11 resulted in dysmorphogenesis, evidenced by decreased crown–rump length, decreased somite number and increased malformation score. During this incubation, we observed elevated HK2 protein concentrations, along with increased glucose consumption and formation of MG. These are hallmark features of HK2-linked unscheduled glycolysis and glycolytic overload [32]. An increased flux of glucose metabolism has long been implicated in hyperglycemia-induced dysmorphogenesis; however, the mechanism of initiation remained unclear [14]. The addition of 10 µM tRES + HESP normalized elevated embryonic HK2 protein levels and reversed glucose-induced embryo dysmorphogenesis, suggesting a functional link of increased HK2 expression and diabetic embryopathy. The elevation of embryonic HK2 protein concentration in high-glucose-concentration cultures was associated with a 75% increase in glucose metabolic flux. This is comparable to the increased glucose metabolism that drives cellular dysfunction in diabetic vascular complications; specific examples include aortal endothelial cells [38,46], retinal pericytes [47], renal mesangial cells [48], proximal tubular epithelial cells [49], and Schwann cells of the peripheral nervous system [50].

**Figure 4:**
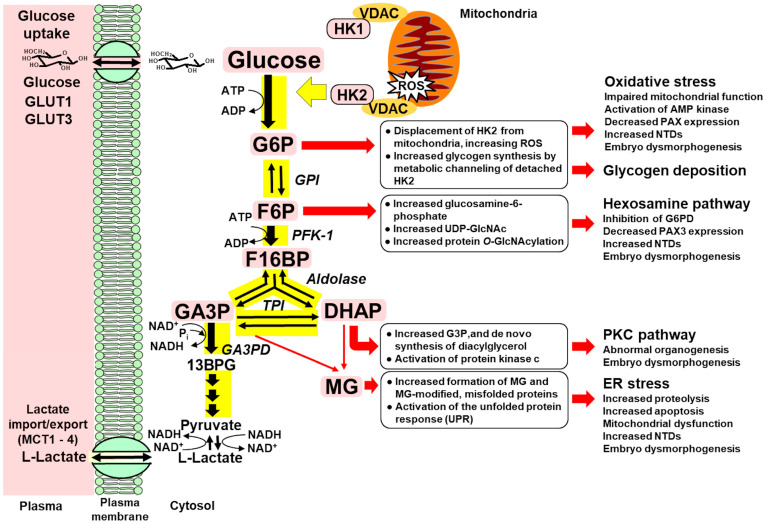
Dysregulation of glycolytic enzymes and metabolic dysfunction in hexokinase-2-linked glycolytic overload hypothesis with impact of effector pathways of metabolic dysfunction and embryo dysmorphogenesis in diabetic embryopathy. Key: Black arrows—metabolism of glucose through early-stage glycolysis; Yellow highlight—HK2 detachment from mitochondria and steps of glycolysis of increased metabolic flux in high glucose concentration; Pink highlighted text—HK2 and metabolites increased in unscheduled glycolysis; and Red arrows—mechanisms of pathogenesis activated in high glucose concentration. Abbreviations: 13BPG, 1,3-bisphosphoglycerate; DHAP, dihydroxyacetonephosphate; F6P, fructose-6-phosphate; F16BP, fructose-1,6-bisphosphate; G6P, glucose-6-phosphate; GA3P, glyceraldehyde-3-phosphate; GA3PD, glyceraldehyde-3-phosphate dehydrogenase; GPI, glucose-6-phosphate isomerase; HK1, hexokinase-1; HK2, hexokinase-2; MCT, monocarboxylate transporter; MG, methylglyoxal; PFK-1, phosphofructokinase-1; ROS, reactive oxygen species; TPI, triosephosphate isomerase; VDAC, voltage-dependent anion channel.

Accordingly, we envisage the development of embryonic metabolic dysfunction in hyperglycemia involving the following responses–described in the HK2-linked glycolytic overload hypothesis [32]. High ambient glucose concentration in early-stage pregnancy produces increased cellular glucose concentration in embryonic cells [34]. This stabilizes HK2 against proteolysis, increasing HK2 protein levels and activity as well as the flux of glucose metabolism without increasing the activity of other early-stage glycolytic enzymes. This increases the steady-state concentration of early-stage glycolytic intermediates, G6P, F6P, fructose-1,6-phosphate (F16BP) and triosephosphates, glyceraldehyde-3-phosphate (GA3P), and dihydroxyacetonephosphate (DHAP), to abnormally high levels. Increased G6P induces a detachment of HK2 from mitochondria, leading to mitochondrial hyperpolarization and increased formation of ROS; this also increases formation and accumulation of embryonic glycogen [32]. Increased formation of ROS contributes to embryo dysmorphogenesis under high glucose concentration, as evidenced by the prevention of abnormalities in crown–rump length, somite number, and malformation score upon the addition of ROS scavengers (superoxide dismutase, catalase, glutathione peroxidase, vitamin E, vitamin C, cysteine derivative [N-acetylcysteine], folic acid, citiolone) to the culture medium [17]. Related induction of oxidative stress by embryonic metabolic dysfunction in hyperglycemia was indicated by increased embryonic isoprostane levels [51], change in the expression of antioxidant response element (ARE)-linked genes [52], decreased activity of glyceraldehyde-3-phosphate dehydrogenase [42], and large-amplitude swelling of embryonic mitochondria, linked to mitochondrial dysfunction [53]. Oxidative stress impairs ATP synthase activity and increases the ADP/ATP ratio activating AMP kinase which decreases expression of the transcription factor, paired box-3 (PAX3), and produces neural tube defects (NTDs)—a common malformation associated with diabetic embryopathy [54]. The low-level accumulation of glycogen in embryos in high glucose concentration [37] is unlikely to be damaging. Increased F6P activates the hexosamine pathway in the following manner: increasing glucosamine-6-phosphate (GlcN6P) and uridine diphosphate-N-acetylglucosamine (UDP-GlcNAc), and O-GlcNAc modification of serine or threonine residues in proteins, O-GlcNAcylation, catalyzed by O-GlcNAc transferase. This has been linked to the inhibition of the pentosephosphate pathway through competitive inhibition of G6PD by GlcN6P, oxidative stress-linked decreased PAX3 expression, and NTDs in diabetic embryopathy [24]. Increased DHAP leads to increased glycerol-3-phosphate and de novo synthesis of diacylglycerol with activation of PKC. Embryonic diacylglycerol and PKC activity was increased by high-glucose exposure on post-conception days 7.5 to 9.5 and days 10 and 11, with increased PKC activity correlated with congenital defects [26,27]. Studies with specific inhibitors implicated PKC-α, -β_2_, and -δ isoforms in embryo malformations [55]. Increased GA3P and DHAP leads to increased formation of MG and increased MG-modified proteins that activate the unfolded protein response (UPR) sensors of the endoplasmic reticulum, inositol requiring enzyme-1α, protein kinase-like ER kinase, and activating transcription factor 6 [56], inducing ER stress. These pathways are activated in the embryo in experimental diabetes, leading to the activation of c-Jun-N-terminal kinase 1/2, increasing apoptosis and proteolysis, mitochondrial dysfunction and NTDs [30,31,57,58]. These pathogenic mechanisms may be concurrently prevented by correcting increased HK2 protein in high glucose concentration with tRES + HESP [38] (Figure 4).

Glycolysis is the major pathway of glucose utilization at this stage of embryogenesis with markedly lower fluxes of pyruvate oxidation and pentosephosphate pathways. This also likely predisposes the embryo to glycolytic overload when exposed to high glucose concentration [59].

Further support for this hypothesis in explanation for embryonic dysmorphogenesis in hyperglycemia came from the effect of the addition of tRES + HESP, which counters HK2-linked unscheduled glycolysis and glycolytic overload in a high glucose concentration [38]. The addition of 5 µM tRES + HESP to embryo cultures with 10 mM glucose decreased HK2 protein concentration by 20%, suggesting a mild glycolytic restriction effect. A similar decrease in glucose consumption (38%) was observed in endothelial cell cultures treated with tRES + HESP under low-glucose concentration conditions [38]. The 42% increase in HK2 protein concentration of embryos in 30 mM glucose cultures is similar to the increase found in endothelial cells and fibroblasts in model hyperglycemia supporting glycolytic overload [38,45]. Correction of the increase in embryonic HK2 protein in high-glucose-concentration cultures by 5–20 µM tRES + HESP suggests that Glo1 inducer treatment may prevent glycolytic overload in the embryo of a mother with diabetes and thus produce a therapeutic response in diabetic embryopathy. The addition of 5 µM tRES + HESP to both low- and high-glucose-concentration cultures increased Glo1 protein concentration, suggesting Glo1 expression was increased as expected for Glo1 inducer activity [39]. However, a high concentration of tRES + HESP (20 µM) decreased embryonic Glo1 protein concentration, suggesting that there were adverse effects of this treatment—also indicated by a decreased somite number and increased malformation score (Figure 2 and Figure 3). The concentrations of tRES and HESP achieved with pharmacologically active doses in vivo do not exceed 10 µM [39,60]; therefore, adverse effects observed at higher concentrations are unlikely to occur in vivo.

We envisage that the protective effect of tRES + HESP against embryo dysmorphogenesis found herein is mediated by the activation of Nrf2 with induction of increased expression of G6PD, Glo1, and other ARE-linked cytoprotective gene expressions [38,39,45,61]. Inducing expression of Glo1 increases the metabolism of MG and glyoxal, and therefore protects against increased advanced glycation end products (AGEs) formed from these reactive dicarbonyl metabolites [39]. Inducing the expression of G6PD decreases G6P and thereby decreases G6P/Mondo A/Mlx-dependent transcription of HK2 to achieve the primary therapeutic response—decreased protein and activity of HK2 [38]. Increasing G6PD activity also has the benefit of diverting metabolic flux to the pentosephosphate pathway, lifting competitive inhibition by GlcN6P and increasing formation of NADPH to counter oxidative stress. The median effective concentration EC_50_ of tRES for Nrf2-dependent induction of Glo1 expression was 1.5 µM in the presence of 5 µM HESP, and EC_50_ of HESP for Nrf2-dependent induction of Glo1 expression was 0.6 µM [39]. The bioavailability of HESP is adequate for the clinical translation of this response where plasma concentrations of ca. 6 µM HESP are readily achieved [62]. The bioavailability of tRES is low but likely sufficient to produce this response [63,64], particularly in combination with HESP, which inhibits human intestinal glucuronosyltransferases and thereby provides for efficient absorption of unconjugated forms of both tRES and HESP [60]. In our previous clinical study of treatment of subjects with overweight and obesity with tRES + HESP, the pharmacological target validation of the Nrf2-dependent induction of increased Glo1 expression and activity was achieved [39]. So, if there is an efficient equilibration of tRES and HESP from blood plasma to embryo, plasma concentrations of tRES and HESP sufficient to activate Nrf2 and induce the therapeutic response for the clinical prevention of diabetic embryopathy may be available. The studies with 5 µM tRES + HESP herein were designed to assess pharmacological activity at concentrations close to the clinical achievable range and 10 µM and 20 µM tRES + HESP to assess the potential for adverse effects at upper outlier concentrations.

Adverse effects of high concentrations of tRES + HESP are likely due to tRES rather than HESP, as identified in a regulatory review of the safety of these compounds [65,66]. At ≤ 10 µM, tRES inhibits phosphodiesterase-1, increasing cAMP [67]. HESP binds and activates protein kinase A (PKA) at a low concentration, 1 µM [68]. Increased cAMP induced by tRES and activation of PKA by HESP synergize to stimulate PKA phosphorylation and activate sirtuin-1, which de-acetylates Nrf2 to maintain its transactivational activity in the cell nucleus. PKA also activates Fyn kinase, which increases translocation of Nrf2 to the cell nucleus to enhance transactivational activity [60,69]. These effects increase the activation of Nrf2 to achieve the therapeutic response [38,39]. At concentrations >10 µM, adverse effects of tRES are activated: inhibition of mitochondrial ATP synthase, which increases the cellular ADP/ATP ratio to activate AMP kinase [70,71], and blocking of the ATP-binding site of phosphoinositide 3-kinase (PI3K), inhibiting PI3K activity [72]. Activation of AMP kinase decreases PAX3 expression, producing NTDs and embryopathy—as occurs in high-glucose-concentration-induced oxidative stress [54] and inhibition of PI3K, which decreases insulin signaling for cell proliferation and maturation in embryo maturation [73]. These mechanisms may explain the decreased protection against the damaging effects of high glucose concentration found with 20 µM tRES in this study.

The mechanistic features of pathogenesis described herein for the rat embryo culture model are also common to embryos of STZ-induced diabetic mice and rats [52,53], mice made transiently hyperglycemic by glucose injection [54], and the 3–4 day blastocyst stage of murine embryos produced by in vitro fertilization and cultured in medium containing 28 mM glucose [74]. For the therapeutic effect of tRES + HESP, tRES (100 mg/kg) partially prevented embryo dysmorphogenesis in STZ diabetic rats [75]. tRES + HESP may be more effective and function at a markedly lower, clinically translatable dose, however, through HESP synergizing with tRES in the activation of Nrf2 and increasing the bioavailability of tRES in vivo [39,60]. Several other pharmacological agents have been found to decrease diabetic embryopathy in experimental models; for example, vinylsulfone [58], quercetin [76] and green tea polyphenol, and epigallocatechin gallate [77]. These compounds are likely too toxic (vinylsulfone) or lack adequate efficacy (quercetin and epigallocatechin gallate) for clinical translation. tRES + HESP has achieved clinical translation for pharmacological target validation, improved glycemic control and correction of insulin resistance in subjects living with overweight and obesity [39]. Unlike previous approaches to therapy, it addresses the initiating mechanism of metabolic dysfunction in hyperglycemia and thereby corrects multiple downstream effector pathways leading to embryo dysmorphogenesis. tRES + HESP is a candidate nutraceutical for further study to prevent diabetic embryopathy.

A key limitation of this study is how therapeutic effects in the embryo culture in the high-glucose-concentration in vitro model translate to experimental in vivo models and to eventually clinical application. We have been impressed by the similarity of the development of day-9 embryos in vivo for 2 days, and in vitro for 48 h, with regard to somites and crown–rump length. Furthermore, the damage caused by maternal diabetes in vivo and 30 mM glucose culture in vitro are also remarkably similar: for example, open neural tube, facial and/or cardiac anomalies, and body rotational defects. We have also found similarities between in vivo and in vitro development in biochemical factors, as well as embryonic gene expression. Adverse effects of treatments are also a major concern where a nutraceutical such as tRES + HESP, particularly a combination enabling a decreased dose of active agents whilst achieving clinical effectiveness, appears to be a reasonable approach towards preventive therapy of diabetic embryopathy. In embryo toxicity studies, compliant with good laboratory practice, with tRES, there were no treatment-related effects at ≥750 mg/kg/day tRES [65]. Similar tests for HESP have not been reported but it is considered a highly safe compound with a median lethal dose LD_50_ > 5000 mg/kg [66]. The clinical dose of tRES and HESP in prospective treatment evaluation with tRES + HESP is ca. 1 mg/kg/day [39]. Nevertheless, before clinical evaluation could be considered, further embryo toxicity studies in laboratory rodents are required for the tRES + HESP combination as well as successful evaluation of the treatment response in animal models of diabetic embryopathy.

Whilst the HK2-linked glycolytic overload hypothesis appears to offer an improved explanation and prospective new route for therapy of hyperglycemia-induced embryo malformations in diabetes, other factors may be involved. The polyol pathway is present in the developing embryo where glucose is metabolized by aldose reductase (AR) to sorbitol and then sorbitol is converted to fructose by sorbitol dehydrogenase. The flux through this pathway is not limited by hexokinase but rather by in situ activity of AR [78]. It likely reflects a minor pathway of glucose metabolism in the embryo. We and others have investigated the possible role of the polyol pathway in diabetic embryopathy. Although the embryonic concentration of sorbitol increased 3–5-fold in an experimental model of diabetic embryopathy, it appeared unrelated to embryo malformations. Studies with rat embryos in cultures and pregnant STZ-induced diabetic rats found that treatments with AR inhibitors corrected the increased sorbitol levels without diminishing the increased rates of embryo malformation [79,80,81]. Taken together, the experimental data provides only limited support for the role of sorbitol accumulation in diabetic embryopathy. Also, experimental studies have suggested embryos may be dependent on external glucosamine and its GLUT2-mediated uptake to support enzymatic glycosylation and pentose phosphate pathway activity [82]. This and other factors may also contribute to the development of diabetic embryopathy.

## 5. Conclusions

Our study suggests high-glucose-concentration-induced metabolic dysfunction driving embryo dysmorphogenesis may be initiated by HK2-linked unscheduled glycolysis and glycolytic overload where embryopathy may be prevented by the Glo1 inducer, tRES + HESP.

## Data Availability

All of the data is contained within the article.
